# Current and Future PET Imaging for Multiple Myeloma

**DOI:** 10.3390/life13081701

**Published:** 2023-08-07

**Authors:** Mariko Ishibashi, Miwako Takahashi, Taiga Yamaya, Yoichi Imai

**Affiliations:** 1 Department of Microbiology and Immunology, Nippon Medical School, Tokyo 113-8602, Japan; mariko-ishibashi@nms.ac.jp; 2 Department of Advanced Nuclear Medicine Sciences, Institute for Quantum Medical Science, National Institutes for Quantum Science and Technology, Chiba 263-8555, Japan; takahashi.miwako@qst.go.jp (M.T.); yamaya.taiga@qst.go.jp (T.Y.); 3 Department of Hematology and Oncology, Dokkyo Medical University, Tochigi 321-0293, Japan

**Keywords:** multiple myeloma, tumor microenvironment, PET, immuno-P, radiotracer, minimal residual disease

## Abstract

Positron emission tomography (PET) is an imaging modality used for the noninvasive assessment of tumor staging and response to therapy. PET with ^18^F labeled fluorodeoxyglucose (^18^F-FDG PET) is widely used to assess the active and inactive lesions in patients with multiple myeloma (MM). Despite the availability of ^18^F-FDG PET for the management of MM, PET imaging is less sensitive than next-generation flow cytometry and sequencing. Therefore, the novel PET radiotracers ^64^Cu-LLP2A, ^68^Ga-pentixafor, and ^89^Zr-daratumumab have been developed to target the cell surface antigens of MM cells. Furthermore, recent studies attempted to visualize the tumor-infiltrating lymphocytes using PET imaging in patients with cancer to investigate their prognostic effect; however, these studies have not yet been performed in MM patients. This review summarizes the recent studies on PET with ^18^F-FDG and novel radiotracers for the detection of MM and the resulting preclinical research using MM mouse models and clinical studies. Novel PET technologies may be useful for developing therapeutic strategies for MM in the future.

## 1. Introduction

Multiple myeloma (MM) is an incurable hematological malignancy characterized by the accumulation of abnormal plasma cells (MM cells) in the bone marrow (BM) [1]. Over several years, almost all cases of MM progress from the precursor states, termed monoclonal gammopathy of undetermined significance (MGUS) [1,2]. The transition from MGUS to MM is caused by multiple genetic mutations, in addition to immunoglobulin heavy-chain translocations and/or hyperdiploidy [3,4,5]. Recently, immune cell profiles by single-cell RNA sequencing analysis have revealed that the immune microenvironments are gradually altered, even in MGUS, due to an increase in regulatory T cells (Tregs) and terminal effector T cells [6,7]. MM progresses rapidly and dramatically through the accumulation of genetic and BM microenvironmental changes [8]. Over the past several years, the treatment options for patients with MM have dramatically changed with the emergence of novel agents, such as immunomodulatory drugs (IMiDs, e.g., lenalidomide and pomalidomide), proteasome inhibitors (e.g., bortezomib, carfilzomib, and ixazomib), and monoclonal antibodies (e.g., elotuzumab, daratumumab, and isatuximab) [1,9,10,11]. These treatments have markedly improved the survival outcomes [1]. Furthermore, many clinical trials of new immunotherapies have been carried out for MM, including on monoclonal antibodies, bispecific antibodies, immune checkpoint inhibitors, and chimeric antigen receptor (CAR) T-cell therapy, to alter the interplay between MM cells and the BM microenvironment [12]. Until recently, the clinical response criteria for anti-MM treatment were based on the assessment of serum-free light chain ratio, serum/urine M-protein, or clonal plasma cells amounting to 5% or less in BM samples [13]. More recently, it has become necessary to assess the minimal residual disease (MRD) in the BM with high sensitivity using individualized treatment monitoring to prevent refractory disease and relapse [14]. MRD is an important prognostic maker that can be determined using allele-specific oligonucleotide polymerase chain reaction (ASO-PCR), multiparameter flow cytometry (MFC), next-generation flow cytometry (NGF), next-generation sequencing (NGS), positron emission tomography with computed tomography (PET/CT), or magnetic resonance imaging (MRI) [15]. In the International Myeloma Working Group (IMWG) criteria updated in 2016, the MRD-negative status was defined as the minimum sensitivity of 1 tumor cell per 1 × 10^5^ normal cells (10^−5^ sensitivity threshold) in the BM by either NGF or NGS [14]. Large-scale meta-analyses have demonstrated that MRD negativity is associated with significant improvements in both progression-free survival (PFS) and overall survival (OS) in the patients with MM [16,17,18,19]. MRD assessments using NGF and NGS allow for the high-sensitivity detection of MRD, but rely on single BM aspirates and might lead to false-negative results due to the heterogeneous distribution of clonal plasma cells in the BM. The current IMWG recommendations define MRD negativity in the BM and whole-body scan negativity using PET/CT [20]. Interestingly, a National Oncologic PET Registry (NOPR) study demonstrated that PET had the greatest impact on MM management in 18 different cancer types [21]. The PET/CT imaging approach may be available not only to detect active tumor lesions but also to determine the efficacy of anti-MM treatments and predict prognostic outcomes. This review focuses on the latest advances in PET/CT imaging in preclinical and clinical studies using MM mouse models.

## 2. Myeloma Mouse Models

Mouse models for MM research represent a useful tool for investigating tumor biology and predicting the effectiveness of novel MM therapeutic strategies. Previously, it was difficult to grow primary human MM cells in mouse bones, which rendered the development of MM models challenging. The emergence of severe combined immunodeficient mice has facilitated the transplantation of human MM cell lines in these mice. Despite the fact that cell proliferation of MM cells can be observed under immunodeficient conditions, these mouse models do not reflect MM progression. Recently, it has become possible to develop MM in mice by genetic engineering or the administration of mineral oil. These MM mouse models mimic the MM pathologies, and the MM cells derived from these models can be passaged in syngeneic mice [22,23]. Preclinical research on PET/CT imaging assessed active and inactive tumor lesions using the aforementioned MM mouse models. Therefore, the MM mouse models will be discussed in this section (Table 1).

### 2.1. Human MM Xenograft Model

Human MM cell line-derived xenograft models are commonly used for the preclinical tests of anti-MM efficacy in vivo. In these models, a variety of human MM cell lines, including U266, MM.1S, OPM2, and RPMI-8226, were implanted subcutaneously or intravenously into immunodeficient mice, including SCID (*Prkdc^scid^*), NOD/SCID (NOD.CB17-*Prkdc^scid^*/J), NOG (NOD.Cg-*Prkdc^scid^Il2rg^tm1Sug^*/ShiJic), and NSG (NOD.Cg-*Prkdc^scid^Il2rg^tm1Wjl^*/SzJ) [22,23]. The engraftment of MM cells is facilitated by the absence of a mouse immune system rejecting the MM cells, and the MM cells grow in the subcutaneous tissue or BM over several weeks. Although these models do not exhibit any MM features, anti-MM efficacy can be assessed in them by measuring changes in the tumor volume.

In contrast, patient-derived primary MM cells cannot grow in immunodeficient mice because their growth and survival are dependent on the support from the human BM microenvironment, including cytokines, growth factors, and complex networks of interactions between MM and other cells [24]. To overcome this, investigators implanted human fetal or rabbit bone chips subcutaneously into SCID mice and human primary MM cells from patients with MM (known as SCID-hu and SCID-rab models, respectively) [25,26,27,28]. Primary MM cells were successively engrafted into mouse models and allowed to expand. To further improve the engraftment rates, Rongvaux et al. generated immunodeficient *Rag2/IL2rg^−/−^* knockout mice with five human knock-in genes encoding macrophage colony-stimulating factor (M-CSF), interleukin-3 (IL-3), granulocyte colony-stimulating factor (G-CSF), signal regulatory protein α (SIRPα), and IL-6, which are important cytokines for innate immune cell and MM cell development [29]. This humanized mouse model was named MIS(KI)TRG6 [29]. When BM cells from patients with MM, MGUS, or asymptomatic MM were injected into the bones of MIS(KI)TRG6 mice, primary MM cells from all patient samples were engrafted into the BM. Furthermore, non-human MM cells, including T, B, natural killer (NK), and myeloid cells can grow in these mice, mimicking the BM microenvironment of the patients with MM [29].

### 2.2. Mineral Oil-Induced Plasmacytoma 315 (MOPC315).BM Mouse Model

Plasmacytomas were experimentally induced by the intraperitoneal injection of mineral oil in BALB/c mice [30]. A MOPC315 cell clone was established in vivo from these mice, which produced IgA monoclonal protein (M protein) [31]. Although this cell clone typically grows subcutaneously in BALB/c mice, Hofgaard et al. transformed the MOPC315 cells into MOPC315.BM cells, which engraft and grow in the BM [32]. To increase the BM tropism of MOPC315 cells, MOPC315.BM cells were established by nine repeated intravenous transplantations of tumor cells engrafted into the femurs of mice [32]. BALB/c mice intravenously transplanted with MOP315.BM cells exhibited human MM-like bone disease. In the MOPC315.BM mouse model, Tregs were induced and accumulated within the BM microenvironment (areas of tumor growth), whereas Treg depletion by the in vivo administration of anti-CD25 slowed the tumor growth [33]. The syngeneic transplantation mouse model allows for the study of the interaction between MM cells and the BM microenvironment, including the immune system, osteoclasts, and other components, and for the assessment of the effect of anti-MM agents in the BM niche.

### 2.3. Spontaneous MM Mouse Model

C57BL/KaLwRiJ mice were reported to spontaneously develop an MGUS-like phenotype and progress to MM with age (5TMM mouse model) [34,35]. This mouse model is characterized by the exhibition of human MM-like diseases, including clonal expansion of malignant plasma cells in the BM, presence of serum IgG2b M protein, disease, renal impairment, and anemia. This mouse MM cell lines 5T2MM and 5T33MM can be passaged in vivo in syngeneic C57BL/KaLwRiJ mice [36,37,38]. The 5T2MM-bearing mice develop osteolytic bone lesions by increasing the receptor activator of nuclear factor-kappa B ligand (RANKL) in the serum, while the 5T33MM-bearing mice do not exhibit bone disease [39]. Furthermore, 5TGM1 cells are a subclone of the 5T33MM cell line established via serial in vivo passaging of 5T33MM cells. The 5TGM1 mouse model developed osteolytic bone lesions [40]. Similar to the MOPC315.BM model, these mouse models are suitable for studying the MM microenvironment. However, their main limitation is their dependence on a particular C57BL/KaLwRiJ mouse strain that is difficult to obtain.

### 2.4. Genetically Engineered Vk*Myc Mouse Model

The Vk*Myc transgenic mouse model is based on a C57BL/6 genetic background and exhibits human MM-like disease due to overexpression of the human MYC transgene, specifically in post-germinal center B cells [41]. The Vk*Myc gene encodes human Myc inserted into the exon sequence of the mouse immunoglobulin kappa (Vk21) gene and harbors a stop codon, TAG, within the Vk21 exon. Therefore, human Myc can be overexpressed by reverting the stop codon (TCG > TAG) via activation-induced cytidine deaminase (AID)-dependent activation triggered by somatic hypermutation [41]. Serum IgG2b M protein and monoclonal MM cell expansion were observed in 80% of Vk*Myc mice of 50 weeks of age. This mouse model develops a more aggressive MM-like disease than the 5TMM mouse model, and the tumor phenotype resembles that of a very aggressive B-cell lymphoma [41]. The Vk12653 and Vk12598 cell lines were generated from aged Vk*myc mice and transplanted in syngeneic mice in vivo [42]. Both cell lines are resistant to bortezomib, but Vk12598 cells showed a better response to melphalan monotreatment than Vk12653 cells [42]. When Vk12653 cells were injected intravenously into Treg-depleted mice, the tumor burden of Vk12653 cells was significantly reduced in the spleen and BM compared to the controls [33]. These mice are B6a.FoxP3.Luci.DTR transgenic mice (C57BL/6 background) that lack Tregs after administration of the diphtheria toxin. Thus, the Vk*Myc mouse model has the advantage that Vk*Myc MM cells can be implanted into transgenic mice with a C57BL/6 genetic background.

**Table 1:** Mouse models of multiple myeloma.

Category	Myeloma Model	Origin	Transplanted Cells	Injection	MM Cell Growth	Reconstruction of Immune System	Bone Disease	References
Xenograft	SCID		Human MM cell lines	Subcutaneous	Subcutaneous	No	No	[22,23]
	NOD/SCID							
	NOG			Subcutaneous Intravenous	Subcutaneous Bone marrow (intravenous)		No (subcutaneous) Yes (intravenous)	
	NSG							
	SCID-hu		Primary MM cells derived from patients with MM	Implanted bone	Within implanted bone	?	Yes (implanted bone)	[25,26,27,28]
	SCID-rab							
	MIS(KI)TRG6 (GM-CSF/SIRPα/IL-3/IL-6 knock-in)		Primary MM cells derived from patients with MGUS/MM	Intrafemoral injection	Bone marrow	Yes (bone marrow)	?	[29]
Syngeneic	MOPC315.BM	BALB/c	MOPC315.BM	Intravenous	Bone marrow Spleen	Yes	Yes	[32]
	5TMM derived model	C57BL/KaLwRiJ	5T2MM	Intravenous	Bone marrow Spleen	Yes	Yes	[34,35,36,37,38,39,40]
			5T33MM				No	
			5TGM1				Yes	
	Vk*Myc derived model	C57BL/6	Vk12598 Vk12653	Intravenous	Bone marrow Spleen	Yes	Yes	[41,42]

## 3. Positron Emission Tomography (PET)

PET is a nuclear medicine imaging technique that can trace the metabolic or biochemical activity of cells in body tissues using positron-emitting isotope-labelled biomolecules (radiotracers) injected into patients. The PET imaging system detects gamma rays produced by positron annihilation events of radiotracers using a ring PET scanner and visualizes active disease in patients. Several positron radionuclides are used as PET radiotracers for research and clinical use in various cancer types, e.g., carbon-11 (^11^C), nitrogen-13 (^13^N), oxygen-15 (^15^O), fluorine-18 (^18^F), copper-64 (^64^Cu), gallium-68 (^68^Ga), bromine-76 (^76^Br), rubidium-82 (^82^Rb), yttrium-86 (^86^Y), zirconium-89 (^89^Zr), and iodine-124 (^124^I; Table 2) [43]. In addition to the assessment of MM activity, PET combined with CT (PET/CT) can monitor the morphological characteristics to detect the presence of lytic lesions, fractures, and extramedullary extensions.

### 3.1. ^18^F-Fluorodeoxyglucose-PET

PET with ^18^F labeled fluorodeoxyglucose (FDG; ^18^F-FDG PET) is widely used for the diagnosis, staging, and the assessment of therapeutic outcomes in patients with cancers, including MM. ^18^F-FDG, a structural analog of glucose, is taken up by cancer cells that are exposed to high glucose levels, allowing the assessment of the metabolic activity in the cancer cells by ^18^F-FDG accumulation. ^18^F decays into stable ^18^O with a mean half-life of 110 min by positron (β^+^) emission (Emax 635 keV), which produces a pair of 511 keV gamma rays (γ) (i.e., annihilation photons) in opposite directions [44]. These gamma rays are detected using a ring PET scanner to visualize ^18^F-FDG-positive lesions in the body. Visual assessment is generally used to interpret PET scans, and positive and negative FDG PETs are defined according to the presence and absence of focal or diffuse lesions of increased FDG uptake above the surrounding background noise in MM, respectively [46]. The standardized uptake value (SUV), which represents the ratio of the tumoral tracer concentration to the average tracer concentration in the whole body, is often used as a semiquantitative measure of the degree of FDG uptake to aid in the interpretation of PET scans [47]. However, the Mayo clinic team reported that the cut-off value of maximum SUV (SUV_max_) was not predictive of PFS or OS in a patient cohort with MM [48]. Currently, it might be difficult to quantitatively assess the sensitivity of PET scans.

Besides ^18^F-FDG PET, whole-body MRI is also used in the assessment of MM. It is a noninvasive imaging technology that is based on the excitation of protons and the detection of the change in the direction of the rotational axis of protons found in the water present in biological tissues [49]. MRI offers excellent contrast resolution for bone and soft tissues, providing high sensitivity for the early detection of focal bone lesions in MM patients [50]. In a meta-analysis of 12 studies, the pooled sensitivity and specificity of ^18^F-FDG PET/CT for MM lesions were 64% (range, 45–79%) and 82% (range, 75–88%), respectively [51]. This analysis reported that the sensitivity of whole-body MRI was higher than that of ^18^F-FDG PET/CT; however, the difference was not significant (*p* = 0.29) [51]. In contrast, ^18^F-FDG PET/CT had greater sensitivity than whole-body MRI (*p* = 0.01) [51]. In other comparative studies, the sensitivity of ^18^F-FDG PET/CT in focal bone lesions was also substantially equal to or slightly lower than that of MRI [52]. On the other hand, ^18^F-FDG PET/CT was reported to have a high sensitivity and specificity of 80–100% in the assessment of extramedullary lesions in MM patients [52]. Another meta-analysis reported that the pooled sensitivity of FDG-PET and PET/CT was significantly higher for the detection of extramedullary lesions than for intramedullary lesions (96% and 61.1%, respectively) [53]. Compared to MRI, ^18^F-FDG PET/CT may miss small or intramedullary lesions; however, ^18^F-FDG PET/CT can distinguish target and non-target lesions with high sensitivity and specificity and has high detection sensitivity for extramedullary lesions in MM patients.

Duncan et al. showed that ^18^F-FDG PET could detect plasma tumor cells in the early, intermediate, and late stages of MM development in a C.IL6Myc mouse model [54]. This MM mouse model is based on human IL-6/mouse c-Myc double-transgenic mice in the mouse strain BALB/c and progresses, developing MM-like neoplasms, by malignant plasma cell transformation [55]. In this mouse model, the ^18^F-FDG PET parameters were used to monitor tumor volume changes and assess refractory disease after the administration of the proteasome inhibitor ixazomib [54]. Thus, it was recently anticipated that ^18^F-FDG PET/CT will be utilized not only to detect active lesions, but also to assess the therapeutic effect of anti-MM agents and patient prognosis. In a retrospective analysis of 195 patients with newly diagnosed MM, ^18^F-FDG PET-negative patients at diagnosis had a significantly prolonged median time to next treatment (TTNT; 55.2 vs. 17.8 months, *p* < 0.0001) and OS (unreached vs. 60.8 months, *p* < 0.0001) than PET-positive patients [56]. Among the patients who achieved a very good partial response (VGPR) or a better response at six months post-treatment, PET-negative patients had a more prolonged OS [56]. Even though the median TTNT and OS were shorter for PET-negative patients in the less-than-VGPR group, these patients also had longer survival times than the positive patients [56]. Thus, the baseline parameters of ^18^F-FDG PET/CT have a strong prognostic value [57].

MRD assessment using NGF or NGS is currently becoming a standard method for assessing the post-treatment prognosis in clinical studies. Both techniques assess MRD with greater sensitivity by analyzing single cells. When undergoing BM assessment 100 days post-autologous hematopoietic stem cell transplant (ASCT), patients with NGS-MRD-negative status at 10^−6^ had longer TTNT than those with MRD negativity at 10^−5^ and positivity, regardless of therapy, cytogenetic risk, and/or R-ISS stage. Almost all NGS-MRD-negative patients were ^18^F-FDG PET-negative [58]. Although MRD assessment shows significantly higher sensitivity than assessment by the current PET imaging, PET imaging provides a considerable benefit over other techniques for assessing lesion extent and disease activity in patients with MM.

### 3.2. New PET Radiotracer

Recently, many new PET radiotracers, other than FDGs, have been explored. These PET assessments accurately identify lesions with peptide- or antibody-based radiotracers that target cell surface antigens on tumor or immune cells.

#### 3.2.1. Peptide-Based Radiotracer

The peptides used as PET radiotracers are high-affinity ligands that target their receptors. One of the candidate targets is very late antigen 4 (VLA4, α4β1 integrin, C49d/CD29), which is highly expressed in MM and BM stromal cells in the tumor microenvironment. LLP2A (molecular formula, C_43_H_54_N_8_O_8_) is a selective peptidomimetic ligand with a high affinity for the activated form of VLA4 [59]. Aberrant VLA4 expression in MM cells enhances cell adhesion-mediated drug resistance by interacting with vascular cell adhesion molecule 1 (VCAM1) expressed in the BM stromal cells. LLP2A was conjugated with the chelators CB-TE1A1P (1,4,8,11-tetraazacyclotetradecane-1-[methane phosphonic acid]-8-[methane carboxylic acid]) and ^64^Cu (^64^Cu-CB-TE1A1P-LLP2A; ^64^Cu-LLP2A) [60]. PET imaging revealed tumor uptake in the spine and femur of NSG mice transplanted intravenously with human MM cell lines 4 h post-injection of ^64^Cu-LLP2A, and further monitoring revealed a decreased tumor size after treatment with bortezomib [61]. The PET signals of ^64^Cu-LLP2A in the MM mouse models correlated with VLA4 expression levels in MM cells. Furthermore, the uptake of ^64^Cu-LLP2A in a VLA4-positive 5TGM1 mouse model was significantly reduced by the pre-administration of unlabeled LLP2A [62]. The first clinical study (NCT03804424) reported that ^64^Cu-LLP2A PET generated a stronger signal than ^18^F-FDG PET in the BM of MM [63]. Interestingly, flow cytometry analysis showed that LLP2A conjugated with the Cy5 dye specifically bound to B, T, and myeloid cells in the BM of 5TGM1-bearing mice, but not in non-tumor-bearing mice [64]. However, VLA4 activated these immune cell subsets in the BM of patients with MM and healthy controls [59,63]. To use ^64^Cu-LLP2A in clinical practice, optimization is required to increase tumor uptake and reduce the background uptake of ^64^Cu-LLP2A in the BM of human subjects.

Another candidate radiotracer is pentixafor (molecular formula, C_60_H_80_N_14_O_14_) conjugated with ^68^Ga via the chelating agent oxodotreotide (DOTATATE; ^68^Ga-DOTATATE-pentixafor, ^68^Ga-pentixafor). Pentixafor is a selective peptidomimetic ligand with a high affinity for chemokine receptor 4 (CXCR4). Although CXCR4 expression is ubiquitous in hematopoietic and non-hematopoietic cells, it is upregulated in MM cells by several tumor microenvironment-related factors, including hypoxia and pro-inflammatory cytokines [65]. CXCR4-expressing MM cells promote tumor growth, survival, drug resistance, migration, and homing by transmitting positive signals through interactions with the C-X-C motif chemokine ligand 12 (CXCL12) expressed on BM stromal cells. The CXCR4-CXCL12 interaction is associated with osteoclastogenesis in the BM of MM. In a clinical study, ^68^Ga-pentixafor PET/CT detected CXCR4-positive disease in 23/35 (65.7%) patients with MM [66]. Of 23 PET-positive patients, 8 patients (34.8%) suffered from intramedullary disease, and 13 patients (56.5%) presented with intra- and extramedullary diseases. In a comparative analysis between ^68^Ga-pentixafor and ^18^F-FDG PET/CT, ^68^Ga-pentixafor had a lower positivity rate than ^18^F-FDG (57.9% vs. 73.7%, respectively) [66]. Another clinical study showed that ^68^Ga-pentixafor and ^18^F-FDG provided a positive signal in 93.3 and 53.3% of the cases, respectively (NCT03436342) [67]. Since CXCR4 is ubiquitously expressed on cells, ^68^Ga-pentixafor PET requires further analysis as regards its uptake background in MM.

#### 3.2.2. Antibody-Based Radiotracers

Immune-based PET imaging (immuno-PET) fuses the exquisite targeting specificity of monoclonal antibodies (mAbs) with the high sensitivity and specificity of whole-body PET imaging. In recent years, investigations on immuno-PET using therapeutic mAbs for treating many different types of cancers have been progressing. In particular, this section provides an overview of radiotracers targeting MM and immune cells.

##### Radiotracers Targeting CD38 Receptor Expression for Imaging MM Cells

Several researchers attempted to evaluate ^89^Zr-desferrioxamine-daratumumab (^89^Zr-DFO-daratumumab, ^89^Zr-daratumumab) for immuno-PET imaging of MM. Daratumumab, an anti-CD38 mAb for the treatment of MM, targets CD38-overexpressing MM and immunosuppressor cells, including regulatory T and B cells (Tregs and Bregs) and myeloid-derived suppressor cells (MDSCs), killing these cells by antibody-dependent cellular cytotoxicity (ADCC), complement-dependent cytotoxicity (CDC), and antibody-dependent cellular phagocytosis (ADCP) [68]. ^89^Zr is a metalloradionuclide with a half-life of 3.3 days and allows immuno-PET imaging to be obtained for up to 6–7 days after its intravenous administration [69,70]. ^89^Zr was conjugated to daratumumab using the DFO chelator. In subcutaneous MM.1S-bearing SCID mouse models, immuno-PET imaging detected radiotracer-incorporated MM tumor masses with volumes ranges of 8.47–128.1 mm^3^ 6–7 days post-administration of ^89^Zr-daratumumab [70]. When MM.1S-bearing mice were injected with pre-administered unlabeled daratumumab as a blocking agent, a radioactivity uptake reduction of ^89^Zr-daratumumab in MM tumors was detected by PET imaging. Tumor uptake within the BM was also observed in NSG mice that were intravenously transplanted with OPM2 [71]. In the first clinical trial (NCT03665155), patients with CD38^+^ MM cells were administered ^89^Zr-daratumumab on day 0 and underwent PET/CT imaging on days 1, 2–4, 5–6, and 7–8 [71]. The radioactivity of ^89^Zr-daratumumab was high in the blood pool and liver 1–2 days post-administration, and its background activity gradually decreased. Conversely, its uptake in focal skeletal lesions consistent with MM showed an increase over time post administration [71]. In addition to ^89^Zr-daratumumab, PET imaging was also performed using daratumumab conjugated with the dodecanetetraacetic acid (DOTA) chelator and ^64^Cu (^64^Cu-DOTA-daratumumab, ^64^Cu-daratumumab), which was extremely stable for up to 48 h in saline solution and mouse serum. PET/CT imaging based on ^64^Cu-daratumumab showed high sensitivity and definitively detected MM tumors in the BM of an MM xenograft model 48 h post administration [72]. In a phase I clinical trial (NCT03311828), PET imaging of ^64^Cu-daratumumab accurately detected sites of MM involvement in patients with MM [73]. Interestingly, ^64^Cu-daratumumab showed higher sensitivity and resolution than ^18^F-FDG for MM tumors in the BM in vivo [72]. A clinical trial comparing ^64^Cu-daratumumab with ^18^F-FDG PET will be conducted in patients with MM.

The Food and Drug Administration (FDA) approved daratumumab and isatuximab as anti-CD38 mAbs for the treatment of patients with MM. Immuno-PET with ^89^Zr-DFO-isatuximab (^89^Zr-isatuximab) detected MM lesions with the same sensitivity as immuno-PET with ^89^Zr-daratumumab in MM.1S-bearing mice [74]. Immuno-PET may be performed in patients with MM treated with daratumumab using ^89^Zr-isatuximab because the epitope of isatuximab does not overlap with the binding site of the CD38 molecule of daratumumab.

##### Radionuclides in Radiotracers for Immuno-PET of MM

^89^Zr and ^64^Cu are currently candidates for radionuclides, and Bally et al. investigated whether either of these radionuclides was the best candidate for immuno-PET of MM [75]. This study was performed by subcutaneous or intravenous administration of anti-mouse CD138 mAb (clone 9E7.4) conjugated with the chelators TE2A-benzyl isothiocyanate (TE2A) and DFO for ^89^Zr and ^64^Cu labeling (^89^Zr-DFO-9E7.4 and ^64^Cu-TE2A-9E7.4, respectively) in the syngeneic 5T33 mouse model. Furthermore, radiotracers were compared with ^18^F-FDG PET imaging. The three radiotracers displayed similar uptake in subcutaneous tumors in the subcutaneous models. In the MM mouse models transplanted intravenously, PET imaging with ^89^Zr-DFO-9E7.4 and ^64^Cu-TE2A-9E7.4 detected tumor uptake with higher sensitivity and specificity than PET with ^18^F-FDG in bone lesions. Because the free ^89^Zr from ^89^Zr-DFO-9E7.4 accumulated in the bone, the tumor-to-bone (background) ratio of ^89^Zr-DFO-9E7 was higher than that of ^64^Cu-TE2A-9E7.4. Weighing these factors alone, ^64^Cu-TE2A-9E7.4 was proposed as the optimal radiotracer for immuno-PET imaging in a preclinical mouse model [75]. Nevertheless, ^89^Zr has the advantages of having a relatively long half-life, a low positron energy, as well as a utility value as that of a radionuclide. The development of better chelator agents for ^89^Zr may be needed to solve the background drawback of free ^89^Zr in PET imaging.

##### Radiotracers Targeting Immune Cells for Imaging the Immune Microenvironment

While PET imaging has been used to visualize tumor lesions in the whole body, recent studies attempted to visualize features of the tumor microenvironment, such as the infiltration status of CD8^+^ T cells. Among various solid tumors, high levels of tumor-infiltrating lymphocytes (TILs) are significantly associated with improved OS and disease-free survival (DFS) compared to low levels [76,77,78,79]. Recently, new types of immunotherapy, such as immune checkpoint inhibitors (ICIs; e.g., anti-PD-1 and anti-PD-L1 mAb), have emerged and been approved for cancer treatment. Programmed death receptor-1 (PD-1) regulates T cell activation by binding to its ligands programmed death ligand 1 (PD-L1) and PD-L2. PD-1 and PD-L1/PD-L2 are highly expressed in TILs and various types of cancers, respectively, and their interactions are associated with anti-immune suppression in the tumor microenvironment [80]. The response rates of these ICIs range from 15–30% for most solid tumors to 40–60% for melanoma and microsatellite instability-high tumors [81], and the differences in the clinical characteristics between responders and non-responder patients to ICIs are not well understood. Kumagai et al. reported that the responders to PD-1 blockade had a higher percentage of PD-1^+^CD8^+^ T cells within the tumors than the non-responders, considering patients with non-small cell lung and gastric cancers [82]. Several studies using immunohistochemistry (IHC), flow cytometry, and time-of-flight (CytoF) mass cytometry have revealed the relationship between TILs and clinical outcomes or the efficacy of ICI treatments. Ruijter et al. demonstrated that immuno-PET using ^89^Zr-labeled CD8-specific one-armed antibody (^89^ZED88082A) detected its accumulation within the tumor lesions in patients with deficiency of mismatch repair (dMMR) tumors, and its accumulation was consistent with the CD8 IHC expression pattern [83]. Furthermore, patients with an above-median baseline level of ^89^ZED88082A uptake had longer PFS and OS than those with an uptake below the median value (*p* = 0.058 and *p* = 0.03, respectively) [83]. Niemeijer et al. performed immuno-PET imaging using the radiotracers ^18^F-FDG, ^18^F-BMS-986192 (^18^F-labeled adnectin with high affinity and specificity for human PD-L1), and ^89^Zr-nivolumab (^89^Zr-labeled anti-human PD-1 mAb) in patients with non-small-cell lung cancer [84]. ^18^F-FDG and ^18^F-BMS-986192 PET scans were obtained 1 h post-injection, and ^89^Zr-nivolumab PET scans were obtained 5–7-day post-injection. ^18^F-BMS-986192 and ^89^Zr-nivolumab PET showed heterogeneous radiotracer uptake in patients with different tumors. The tumor uptakes of ^18^F-BMS-986192 and ^89^Zr-nivolumab were positively correlated. In addition, responders with ≥30% reduction of tumor size 12 weeks post-administration of nivolumab had higher uptake of ^18^F-BMS-986192 and ^89^Zr-nivolumab than non-responders [84].

Preclinical studies of immuno-PET using CD8- or PD-1-targeting radiotracer have not yet been reported for MM. Similar to other cancers, PD-L1 is highly expressed on MM cells, and exhausted PD-1^+^CD8^+^ T cells have increased levels in the BM of patients with MM compared to healthy controls [85,86]. However, the distribution of PD-1^+^CD8^+^ T cells in the BM microenvironment of MM is not well understood, and their prognostic relevance is unknown. To clarify these points, immuno-PET is necessary to assess the T cell dynamics in the tumor microenvironment of MM using a syngeneic myeloma mouse model with a maintained immune system.

## 4. Conclusions

PET is the best noninvasive approach for assessing the distribution of disease lesions and the response to treatment. PET imaging using several novel radiotracers has been performed in clinical and preclinical studies of MM (Table 3). Additionally, an analysis comparing the sensitivity of PET imaging using ^18^F-FDG and novel radiotracers was performed.

Aggressive late-stage MM (stage III) exhibits elevated glucose uptake, which is evident from the increased PET positivity, whereas early-stage MM (stages I and II) is PET-negative due to its reduced glucose uptake [87]. Currently, metabolic active sites, such as extramedullary lesions, as well as bone damage in aggressive late-stage MM can be detected. The recent developments in PET imaging and the discovery of radiotracers having higher sensitivity and specificity than those previously used should increase the clinical utility and value of imaging. Specific radiotracers that target the cell surface antigens of MM cells, such as ^89^Zr-daratumumab, ^64^Cu-daratumumab, ^64^Cu-LLP2A, and ^68^Ga-pentixafor, detected intramedullary lesions of MM with high sensitivity and specificity compared to ^18^F-FDG. Thus, antigen-specific radiotracers may be advantageous over non-specific radiotracers for MM detection. To detect MM lesions more specifically, it is necessary to identify new MM-related targets and the corresponding target-specific agents. Furthermore, the optimization of PET radiotracers is critical for alleviating their background uptake. Despite the availability of these imaging techniques for the management of MM, the sensitivity of PET imaging is not comparable to that of MRD negativity at the 10^−5^ threshold for NGF and NGS. To overcome this challenge, whole-gamma imaging (WGI) is currently being developed as next-generation PET. WGI comprises PET combined with a Compton camera by inserting a scanner ring into a PET ring, which will provide PET information with high sensitivity and resolution in the future [88]. To increase the value of PET in assessing the treatment response and prognosis in MM patients, the development of sensitive and specific PET with new radiotracers must be further carried out.

Recently, immune-based therapies have played an increasingly important role in the mainstream treatment of MM. Thus, to understand the immune dynamics in the BM microenvironment pre- and post-treatment, immuno-PET with immune cell-targeted radiotracers is a necessary evaluation tool for the assessment of prognosis and the immunotherapeutic response. This immuno-PET platform, in combination with ^18^F-FDG, should be developed and evaluated using MM mouse models with intact immune systems. Novel PET technologies may be promising tools for the development of therapeutic strategies for MM.

## Figures and Tables

**Table 2:** Radionuclides in PET imaging used for cancer diagnosis.

Radionuclide	Abbreviation	Emission Type	Half-Life	PET Radiotracers in Various Cancer Types
Carbon-11	^11^C	β+	20.4 min	^11^C-choline, ^11^C-acetate, ^11^C-methionine
Nitrogen-13	^13^N	β+	10.0 min	^13^N-ammonia
Oxygen-15	^15^O	β+	2.0 min	^15^O-oxygen
Fluorine-18	^18^F	β+	110 min	^18^F-FDG, ^18^F-FET, ^18^F-fluorocholine, ^18^F-fluoride
Copper-64	^64^Cu	β+	12.7 h	^64^Cu-LLP2A, ^64^Cu-pembrolizumab, ^64^Cu-pentixafor, ^64^Cu-Rituximab, ^64^Cu-Bombesin, ^64^Cu-Trastuzumab
Zirconium-89	^89^Zr	β+	78.4 h	^89^Zr-Daratumumab, ^89^Zr-Trastuzumab, ^89^Zr-atezolizumab, ^89^Zr-bevacizumab, ^89^Zr-girentuximab
Gallium-68	^68^Ga	β+/γ	67.8 min	^68^Ga-pentixafor, ^68^Ga-FAPI, ^68^Ga-PSMA, ^68^Ga-GRP
Bromine-76	^76^Br	β+/γ	16.2 h	
Rubidium-82	^82^Rb	β+/γ	1.3 min	
Yttrium-86	^86^Y	β+/γ	14.7 h	
Iodine-124	^124^I	β+/γ	100.2 h	^123^I-Iodometomidate, ^123^I-MIBG

Note: Information is cited from Coniti M, et al. (reference [44]), Rong J, et al. (reference [45]), and ClinicalTrials.gov (https://beta.clinicaltrials.gov/) accessed on 1 June 2023. Abbreviations: FDG, fluorodeoxyglucose; FET, fluoroethyltyrosine; FAPI, fibroblast activation protein inhibitor; PSMA, prostate-specific membrane antigen; GRP, gastrin-releasing peptide; MIBG, meta-iodobenzylguanidine.

**Table 3:** The PET radiotracers used in clinical and preclinical studies on MM.

Tracer Type	Radiotracer	Target/Mechanism	Phase	NCT Number
Unspecific Tracer	^18^F-fluciclovine	Amino acid metabolism	Not Applicable	NCT03966443
	^18^F-choline	Cell membrane synthesis	Phase 3	NCT03891914
	^11^C-acetate	Fatty acid metabolism	Phase 2	NCT03262389
	^11^C-methionine	Amino acid metabolism		
	^18^F-fluorocholine	Lipid metabolism	Not Applicable	NCT04349358
	^18^F-fludarabine	Purine nucleoside analog	Phase 1	NCT03832127
Specific Tracer	^64^Cu-LLP2A	VLA4-targeted ligand	Early phase 1	NCT03804424
	^68^Ga-pentixafor	CXCR4-targeted ligand	Early phase 1 Early phase 1 Early phase 1 Phase 2	NCT03436342 NCT05364177 NCT05093335 NCT04561492
	^68^Ga-pentixather	CXCR4-targeted ligand	Early phase 1	NCT05364177
	^18^F-PSMA-1007	Prostate specific membrane antigen (PSMA)-targeted ligand	Not Applicable	NCT05448404
	^18^F-tetrafluoroborate (BF4)	Sodium/iodide symporter (NIS)-targeted ligand	phae1/2	NCT02907073
	^89^Zr-daratumumab	Anti-CD38 antibody	Phae1/2 Phase2 Phase 2	NCT03665155 NCT04467281 NCT04814615
	^64^Cu-daratumumab	Anti-CD38 antibody	Phase 1	NCT03311828
	^89^Zr-satuximab	Anti-CD38 antibody		
	^68^Ga-Nb1053	CD38-specific single domain antibody (Nb1053)		
	^89^Zr-elotuzumab	Anti-SLAMF7 antibody		
	^89^Zr-bevacizumab	Anti-VEGF antibody	Not Applicable	NCT01859234

Note: Information is cited from ClinicalTrials.gov (https://beta.clinicaltrials.gov/) accessed on 1 Jun 2023.

## Data Availability

The data presented in this review are available upon request to the corresponding author.
